# A case of envenomation by the false fer-de-lance snake *Leptodeira annulata* (Linnaeus, 1758) in the department of La Guajira, Colombia

**DOI:** 10.7705/biomedica.4773

**Published:** 2020-03-30

**Authors:** Teddy Angarita-Sierra, Alejandro Montañez-Méndez, Tatiana Toro-Sánchez, Ariadna Rodríguez-Vargas

**Affiliations:** 1 Vicerrectoría de Investigación, Universidad Manuela Beltrán, Bogotá, D.C., Colombia Universidad Manuela Beltrán Universidad Manuela Beltrán BogotáD.C Colombia; 2 Yoluka ONG, Fundación de Investigación en Biodiversidad y Conservación, Bogotá, D.C., Colombia Fundación de Investigación en Biodiversidad y Conservación BogotáD.C Colombia; 3 Grupo de Investigación en Proteínas, Departamento de Química, Universidad Nacional de Colombia, Bogotá, D.C., Colombia Universidad Nacional de Colombia Grupo de Investigación en Proteínas Departamento de Química Universidad Nacional de Colombia BogotáD.C Colombia

**Keywords:** Colubridae, snake bites, edema, poisons, Colombia, Colubridae, mordeduras de serpiente, edema, venenos, Colombia

## Abstract

Envenomations by colubrid snakes in Colombia are poorly known, consequently, the clinical relevance of these species in snakebite accidents has been historically underestimated. Herein, we report the first case of envenomation by opisthoglyphous snakes in Colombia occurred under fieldwork conditions at the municipality of Distracción, in the department of La Guajira. A female biologist was bitten on the index finger knuckle of her right hand when she tried to handle a false fer-de-lance snake *(Leptodeira annulata).* Ten minutes after the snakebite, the patient started to have symptoms of mild local envenomation such as edema, itching, and pain in the wound. After 40 minutes, the edema reached its maximum extension covering the dorsal surface of the right hand and causing complete loss of mobility. The clinical treatment focused on pain and swelling control. No laboratory tests were performed. The patient showed good progress with the total regression of the edema 120 hours after the snake-bite accident and complete recovery of the movement of the limb in one week. Venomous bites of "non-venomous snakes" (opisthoglyphous colubrid snakes) must be considered as a significant public health problem because patients lose their work capability during hours or even days and they are forced to seek medical assistance to treat the envenomation manifestations.

## Resumen

Los envenenamientos por colúbridos en Colombia son poco conocidos, por lo que la relevancia clínica de estas especies de serpientes en los accidentes ofídicos ha sido históricamente subestimada. Se presenta el primer caso de envenenamiento por una serpiente opistoglifa en Colombia, ocurrido bajo condiciones de trabajo de campo en el municipio de Distracción, departamento de La Guajira.

Una bióloga fue mordida en el nudillo del dedo índice de la mano derecha cuando trató de manipular una falsa cabeza de lanza o falsa mapanare *(Leptodeira annulata).* Diez minutos después de la mordedura de la serpiente, la paciente presentó síntomas de envenenamiento local leve, como edema, picazón y dolor en la herida. Después de 40 minutos, el edema alcanzó su extensión máxima, cubriendo la superficie dorsal de la mano derecha y causando la pérdida completa de la movilidad. El tratamiento clínico se centró en el control del dolor y la inflamación. No se hicieron pruebas de laboratorio. La paciente tuvo una buena evolución, con la regresión total del edema 120 horas después del accidente ofídico y la normalización de los movimientos de la extremidad a la semana. Las mordeduras venenosas de serpientes "no venenosas" (serpientes colúbridas opistoglifas) deben considerarse un problema de salud pública importante, ya que los pacientes pierden la capacidad de trabajo durante horas o días y se ven obligados a buscar asistencia médica para contrarrestar las manifestaciones locales de envenenamiento.

Snakebite accidents are a serious health issue in tropical regions, particularly in rural and suburban areas. Among South American countries, Colombia ranks third in the number of snakebites per year (~4,750), and sixth in snakebite incidents per 100,000 inhabitants (~9.1) ^1^^,^^2^. Clinically important cases of envenomation by snakes are most often caused by bites of viperid and elapid species ^3^. Thus, the clinical relevance of colubrid species in snakebite accidents have been underestimated, as evidenced by the number of lethal cases of envenomation reported, the undetermined percentage of colubrid snakes that secrete toxins from an apparatus capable to generate envenomation, the improper taxonomic identification of the snakes responsible for the accident, and the ineffective surveillance systems in tropical countries where reporting cases of envenomation by colubrids is not required ^4^.

The number of snake species in Colombia surpasses 300 and only 18 % (~24 viperid species, ~31 elapid species) of them are potentially dangerous for people ^5^^,^^6^. This means that most of the Colombian ophidian fauna is composed of harmless colubrid snakes (~187 species). Nevertheless, several snakes from the Colubridae family are considered non-venomous although they have an opisthoglyphous (rear-fanged) dentition and their venom has mild or high toxicity ^4^^,^^7^.

*Leptodeira annulata* (Linnaeus, 1758) is one of these opisthoglyphous colubrid snakes which have a broad distribution including western Panamá, Colombia, Ecuador, Venezuela, Trinidad and Tobago, and Brazil at altitudes between sea level and 1,000 masl ^8^.

In Colombia, the species has a widespread distribution including the Amazon and Chocó rainforests, the evergreen forest along the main Andean rivers, the Orinoco savannas, and the xerophytic forest in the Caribbean coast ^6^^,^^8^.

This species feeds mainly on small frogs and lizards that are killed by the injection of venom with proteolytic activity ^9^. These snakes have nocturnal activity, semi-arboreal habits, oviparous reproductive mode, and they inhabit several types of tropical habitats ^8^^-^^10^.

Herein we describe a snake-bite accident caused by *L. annulata* to a female biologist under fieldwork conditions in La Guajira department, Colombia.

## Case report

**Figure 1:**
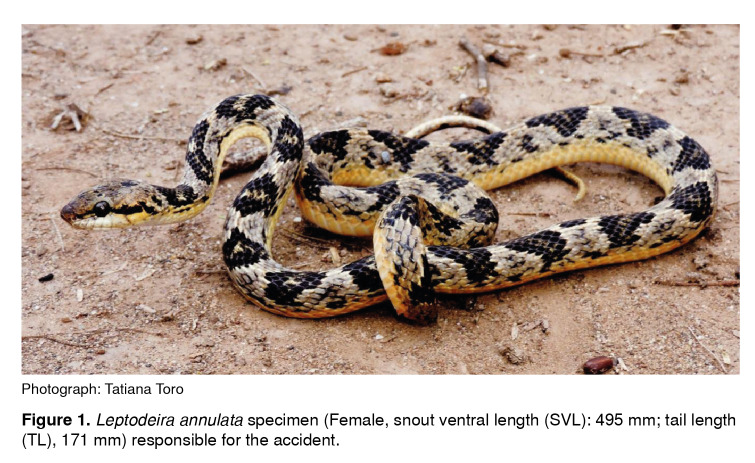
*Leptodeira annulata* specimen (Female, snout ventral length (SVL): 495 mm; tail length (TL), 171 mm) responsible for the accident.

**Figure 2:**
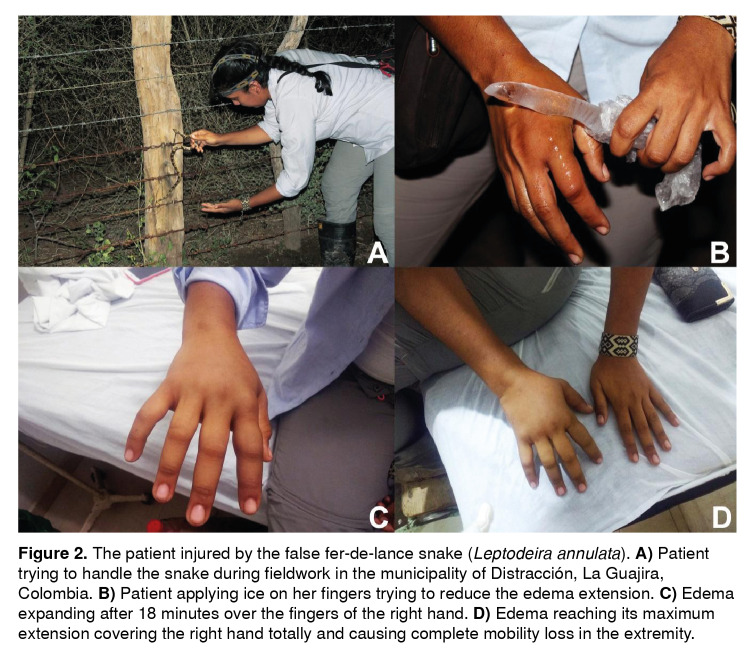
The patient injured by the false fer-de-lance snake *(Leptodeira annulata).* **A)** Patient trying to handle the snake during fieldwork in the municipality of Distracción, La Guajira, Colombia. **B)** Patient applying ice on her fingers trying to reduce the edema extension. **C)** Edema expanding after 18 minutes over the fingers of the right hand. **D)** Edema reaching its maximum extension covering the right hand totally and causing complete mobility loss in the extremity.

On April 27, 2018, at 19:20 hours, a 29-year old female biologist was bitten by a false fer-de-lance snake (L. *annulata,* male, snout ventral length (SVL)=495 mm, tail length (TL)=171 mm) (figure 1) on the knuckle of the second digit of her right hand when she was trying to catch the snake during fieldwork at the municipality of Distracción, La Guajira department, Colombia (figure 2A).

Photograph: Tatiana Toro

The snake remained attached to the knuckle for about 10 seconds until she asked for help. Once her fieldwork partner detached the snake from the knuckle, the patient had a burning sensation and itching in the site of the bite, which vanished 3 minutes later, but the pain remained. The bite marks were evident in the injury a few minutes after the bite. After 12 minutes, she noticed the emergence of mild edema at the affected site and she applied water and iodized antiseptic on the wound. After 18 minutes, the edema spread towards the rest of the fingers. The patient applied ice on her fingers directly on the skin trying to reduce the extension of the edema (figure 2B), but this had no effect against the growing edema (figure 2C). After 40 minutes, the edema reached its maximum extension covering the dorsal surface of the hand and causing complete mobility loss in the fingers and the hand (figure 2D). No tourniquet was used.

After 95 minutes of the snake bite accident, the patient received medical care at the emergency service of the *Hospital San Agustín* in the municipality of Fonseca. She was conscious, oriented, and afebrile and referred mild pain at the affected site, numbness, and difficulty in mobilizing the fingers of her right hand. No systemic symptoms were referred.

During the physical examination, the patient had mild hypertension (130/90 mm Hg), however, the rest of her vital signs were stable. There were signs of edema involving fingers, the dorsal surface of the right hand, and the distal third of the ipsilateral forearm. Appropriate mobility was restricted by the edema in the affected regions. There was no evidence of hemorrhagic blistering, ecchymosis, angioedema, lymphadenopathies or other relevant signs. The patient had no significant medical or surgical history.

The wound was washed again with antiseptics and clinical supervision was based on symptomatic management: Pain, swelling, and potential infection control with intravenous sodium chloride solution, a single intravenous dose of hydrocortisone (400 mg), a single intravenous dose of ceftriaxone (1 g) and tetanus vaccine. The patient was under observation for approximately 12 hours during which her blood pressure normalized, edema and pain decreased, and she had moderate recovery of her mobility.

The snake was shown to the medical practitioner on duty; however, she could not establish the taxonomic identity of the snake or whether the specimen was venomous. Hence, the patient and her fieldwork partner explained to the medical practitioner that the accident was not a bothropic envenomation and, therefore, no antivenom was applied.

The patient was discharged with a medical prescription of desloratadine, 5 mg, orally twice a day for 7 days; ciprofloxacin, 500 mg, orally every 8 hours for 7 days, and naproxen, 500 mg, orally three times a day for 7 days; however, she did not follow it. The edema on the right hand disappeared 120 hours after the snake bite accident and the patient recovered the complete mobility of her extremity one week later. Mild pain at the bitten site continued for eight more days.

### Ethical considerations

The study complies with Colombian Resolutions 84/1989, 14/1986, and 3/2001, as well as with the Universal Declaration on Animal Welfare (UDAW) adopted by the United Nations in 2011. The third author of the study was the patient bitten by the false fer-de-lance snake *(Leptodeira annulata)* and she signed an informed consent.

## Discussion

Among the more than 780 Neotropical snake species, less than 5% (~35 species) of them have been reported in cases of envenomation: *Apostolepis* spp., *Borikenophis portoricensis, Chiroius* spp., *Clelia plumbea, Coniophanes imperialis, Conophis lineatus, C. vittatus, Crisantophis nevermanni, Cubophis cantherigerus, Erythrolamprus aesculapii, E. bizona, E. miliaris, E. poecilogyrus, Helicops angulatus, H. tapajonicus, Hydrodynastes gigas, Leptophis ahaetulla, L. diplotropis, Leptodeira annulata, L septemtrionalis, Mastigodryas* spp., *Oxybelis aneus, Philodryas baroni, P. olfersi, P. patagoniensis, Urotheca elapiodes, Sibynomorphus mikanii, Symphimus* spp., *Thamnodynastes pallidus, T. strigatus, Tomodon dorsatus, Tropidodryas* spp., *Xenodon merremii* and *Xenodon severus*^4^.

*Leptodeira annulata* has been documented only in two previous cases of envenomation with mild local effects, as in the present case of envenomation ^11^^,^^12^, although the venom of *L. annulata* has shown high proteolytic and hemorrhagic activity in experimental studies in rats ^13^. According to reports ^13^^,^^14^, possibly the growing edema and the complete mobility loss of the fingers and hand observed in our patient were mediated by the metalloproteinase activity associated with the inflammatory mediators.

The clinical manifestations in the present case of envenomation by *L. annulata,* as well as in previous cases reported, agree with most of those described in the accidents caused by colubrid species, which include mild pain, edema, erythema, and transient bleeding, the latter possibly due to the mechanical effect of the bite ^15^. Also, progressive coagulopathy and hemorrhagic diathesis can be complicated by acute kidney injury in rare cases ^16^. However, the relationship between the colubrid venom and the elevated blood pressure observed in the patient is unclear. There are few reports about transient hypertension in other colubrid envenomation ^17^, nevertheless, blood pressure and renal function monitoring in these patients is recommended.

Traditionally, medical treatment includes supportive therapy with antihistamines and analgesics. Only a few patients receive treatment with viperid antivenom, which is not recommended because its neutralizing effect is not demonstrated and the patient can be exposed to suffer severe adverse reactions after the administration of heterologous sera ^14^^,^^18^. Although some reports indicate the use of NSAID, antihistamines, and corticosteroids, there is no controlled clinical study on its usefulness in such a situation. In contrast, the administration of tetanus vaccine is recommended ^15^. In the cases of accidents due to venomous snakes, which may cause platelet dysfunction, coagulopathy, and hemorrhage syndrome, it has been found that the use of NSAID seems to be safe, as long as they do not contribute to worsening the hemorrhagic effects ^19^.

It has been suggested that in snakebite accidents caused by colubrids, the seriousness of the envenomation may depend on the time the snake attaches to the flesh or with multiple bites in the injury area ^20^. Therefore, the snake should be detached from the bitten area as fast as possible, as this contributes to decreasing the chance of toxin release and breakthrough into the tissues. Likewise, in accidents caused by colubrids, even in accidents by viperid or elapid snakes, the secondary infection of the wound occurs in around 1% of the cases, likely due to the antimicrobial effect of the venom, which explains why no prophylactic antibiotics are used ^21^. The use of antibiotics should be supported in microbiological analyses. It is assumed that the oral cavity of the snake can contain a great diversity of Gram-positive and Gram-negative bacteria. Gram-negative microorganisms are susceptible to imipenem and levofloxacin while Gram-positive are susceptible to azithromycin and amoxicillin/clavulanate ^22^.

*Leptodeira annulata* is not considered an aggressive snake ^9^. It follows the usual antipredator behavior pattern of hierarchical decision-making observed in several venomous and non-venomous snakes ^23^^,^^24^: First, if a predator stimulus is detected, the reaction is to retreat (escape behavior); second, if the threatening stimulus persists, the reaction is to employ passive deterrents (head hide, body coiled, crouching, immobility, ball position), and third, if the threat further escalates, the reaction is to engage in aggressive defense (dorsoventral neck compression, head compression, jump, or bite). Probably, in the present case, the patient was not aware of antipredator behaviors displayed by the snake that could have alerted her about the stress caused on the snake by her handling. Thus, when the snake displayed aggressive defensive behavior she could not anticipate the strike and, as a result, the snakebite accident occurred.

During the period 2013-2018, the annual mean of snakebite accidents in Colombia was 4,160 cases, of which those caused by colubrid snakes ranged from 33 to 128 cases per year ^1^^,^^25^. However, in the same period, in more than 1,206 cases each year (~29% of the annual mean of snake-bite accidents) ^25^ there was no accurate identification of the snake responsible for the accident suggesting that their number may be underestimated. It has been pointed out that one of the main factors that cause inappropriate treatment of snakebite accidents in Colombia is the misidentification or lack of identification of the snake responsible for the accident by the medical practitioner or the medical staff treating the patient ^6^. Therefore, training the medical staff on snakes potentially dangerous for people, as well as the generation of tools to facilitate diagnose and implement appropriate treatments, must be a priority.

We report here the first case of envenomation by opisthoglyphous snakes in Colombia, which supports the idea that venomous bites of non-venomous snakes (opisthoglyphous colubrid snakes) must be considered as a significant public health problem, as patients lose work capability during hours or days and they are forced to seek medical assistance to counter envenomation manifestations ^4^^,^^18^^,^^20^. The recommended medical treatment starts by washing the area with water and soap, as well as local antiseptics. The use of tourniquets or the practice of suction, incisions or local administration of other substances is not indicated ^14^. The management is predominantly symptomatic with strict monitoring of coagulation times, since transient alterations may occur ^18^.
